# Sediment vibration characteristics based on accelerometer measurements

**DOI:** 10.1038/s41598-023-28209-8

**Published:** 2023-01-21

**Authors:** Pingyi Wang, Jun Yuan, Meili Wang, Mi Wang

**Affiliations:** 1 College of Hehai, Chongqing Jiaotong University, Chongqing, 400074 China; 2 School of Transportation and Municipal Engineering, Chongqing Jianzhu College, Chongqing, 400072 China

**Keywords:** Fluid dynamics, Statistical physics, thermodynamics and nonlinear dynamics

## Abstract

The study of the vibration phenomenon of pebbles under turbulence is still a gap despite recent technological advancements in measurement capabilities. In this study, the vibration process of a fully exposed, isolated smart pebble on a rough bed was measured using a miniature inertial accelerometer and combined with simultaneous local measurements of the near-bed velocities. The paper conducts a series of experimental studies with different flow conditions. The test data match well with the manually observed phenomena, indicating the authenticity of the sediment vibration data collected by the measurement system. The test results show that the pebble motion (before entrainment) subjected to turbulence is a nonlinear vibration process, and its vibration types include in-situ vibration and ex-situ strong vibration. The probability distribution for the amplitude of vibration acceleration is well parameterized by the normal distribution. The vibration intensity tends to increase before approaching the threshold, but it weakens when approaching the point. The sediment vibration frequency is within 20–25 Hz, similar to the flow pulsation frequency and belongs to low-frequency vibration. The data indicate that the near-bed flow velocity is most directly related to the particle vibration events.

## Introduction

The incipient motion of sediment particles is a complex stochastic phenomenon. The motion conditions determine the stability of erodible riverbeds, constituting the most complicated problem of sediment transport in rivers, estuaries, and coastal areas. Although much research has been done on sediment entrainment, there is still no widely accepted model to explain the sediment transport problem^1^. Shields^2^ first proposed that individual particles move under a time-averaged mean bed shear stress, while Varenius^3^ highlighted the importance of turbulence on particle movement. Shields’ criterion has been widely criticized for its ambiguity and limited applicability^4–7^, and the validity of a single standard or even the existence of measurable critical threshold conditions has been questioned^8^. Varenius concluded from his observations that the random movement of sediment particles is related to the fluctuating motion of water. Many researchers have advocated this point of view based on detailed field, laboratory, and other studies^9–13^. Since then, related scholars have proposed new interpretations, such as impulse, work, and energy, to overcome the limitations of the formulation based on the time-averaged Shields parameter and thus improve the prediction of the initiation of sediment particle movement^14–19^. The primary purpose of these efforts is to relate the fluctuating nature of turbulent flows with the "energy barrier"^17^ that a particle needs to overcome.

Early research models have used hydrostatic theory (forces, moments, impulses) to analyze sediment entrainment problems. However, field and laboratory observations show that some particles vibrate or rock before entrainment^15,16,20,21^, and near the threshold, entrainment events occur under the influence of local turbulence. Therefore, the previous research results did not reveal the motion mechanism of sediment entrainment. Sediment entrainment does not belong to the category of statics, but to nonlinear dynamics. Some scholars use the mutation theory to explain the sediment entrainment problem^22^. Based on the nonlinear theory^23,24^, the researchers established the cusp mutation model of non-uniform sediment entrainment^25,26^, derived the critical condition equation of sediment entrainment^27,28^, and constructed the non-linear sand transport rate equation^29^. The existing research results, which introduce more nonlinear modes, stay in theoretical analysis and do not conduct experimental research. And the monitoring of the real sediment entrainment process is still blank. Therefore, collecting vibration data of sediment entrainment process is beneficial to reveal the motion mechanism of sediment entrainment. Analyzing the vibration characteristics of sediment under the action of turbulence can improve the prediction of sediment motion initiation.

The intermittent motion characteristics of bed sediment and the randomness of particle motion forms make the experimental observation of sediment entrainment very difficult. At present, experimental measurement techniques such as underwater photography (UP) and high-speed imaging are used to observe and measure the phenomenon of particle motion. However, the vibration data during sediment entrainment cannot be measured by traditional methods, and new monitoring methods need to be explored. In recent years, the miniaturization of sensing equipment has made the concept of a smart pebble^21,30–35^ (a small, free-moving multisensor capable of measuring inertial dynamics such as acceleration and angular velocity) feasible. Maniatis proposed a new method to approximate the probability of individual coarse particle entrainment display^21^, and then measured the inertial drag and lift of coarse particles on a rough alluvial layer using a particle accelerometer^35^. Oliver Gronz^34^ used a small nine-axis sensor implanted in a stone to track the movement of the pebbles. Smart pebbles have measured the sediment transport process, but the sediment vibration process has not been monitored.

Therefore, this paper selects micro acceleration sensor to measure the vibration process of sediment entrainment, which has the following advantages compared with the traditional measurement methods:

1. Underwater photography (UP), high-speed imaging techniques can measure the motion parameters during particle entrainment. However, in most cases the presence of large amounts of tiny sediment and air bubbles in the water makes the current turbid, making the instrument unable to discern the movement of pebbles. In contrast, acceleration sensors are unaffected by the turbidity of the water.
2. The traditional method cannot measure the vibration data when the sediment is entrained, but the accelerometer can collect the three-axis acceleration when the sediment is vibrating.
3. The acceleration sensor has a resolution of 0.1 mg and a sampling frequency of 200 Hz, so the measurement accuracy can reflect the vibration of sediment entrainment.
4. The existing smart pebble technology has a large sensor size (d = 4 cm), while the miniature acceleration sensor customized in this paper is smaller in size and can measure the movement of small grain size sediment.

To study the mechanism of sediment vibration, this paper is the first to explore the method of monitoring the natural sediment vibration process using micro-accelerometer. The vibration signal of a fully exposed, isolated natural pebble was collected through a laboratory flume experiment, and the local water velocity was measured simultaneously using an acoustic Doppler velocimeter (ADV). Explore the relationship between near-bed flow velocity and pebble vibration, and analyze the pebble vibration characteristics (vibration intensity, vibration frequency).

## Methods

The experiment was designed so that pebble vibrations could be more easily identified and correlated with simultaneously measured near-bed velocities. In this study, to simplify the phenomenon to its most elemental form to facilitate the development of cause and effect relations (while retaining the physics which dominates), the vibration of an isolated, fully exposed, natural particle placed on a rough bed was examined.

### Design of Smart pebble

**Figure 1:**
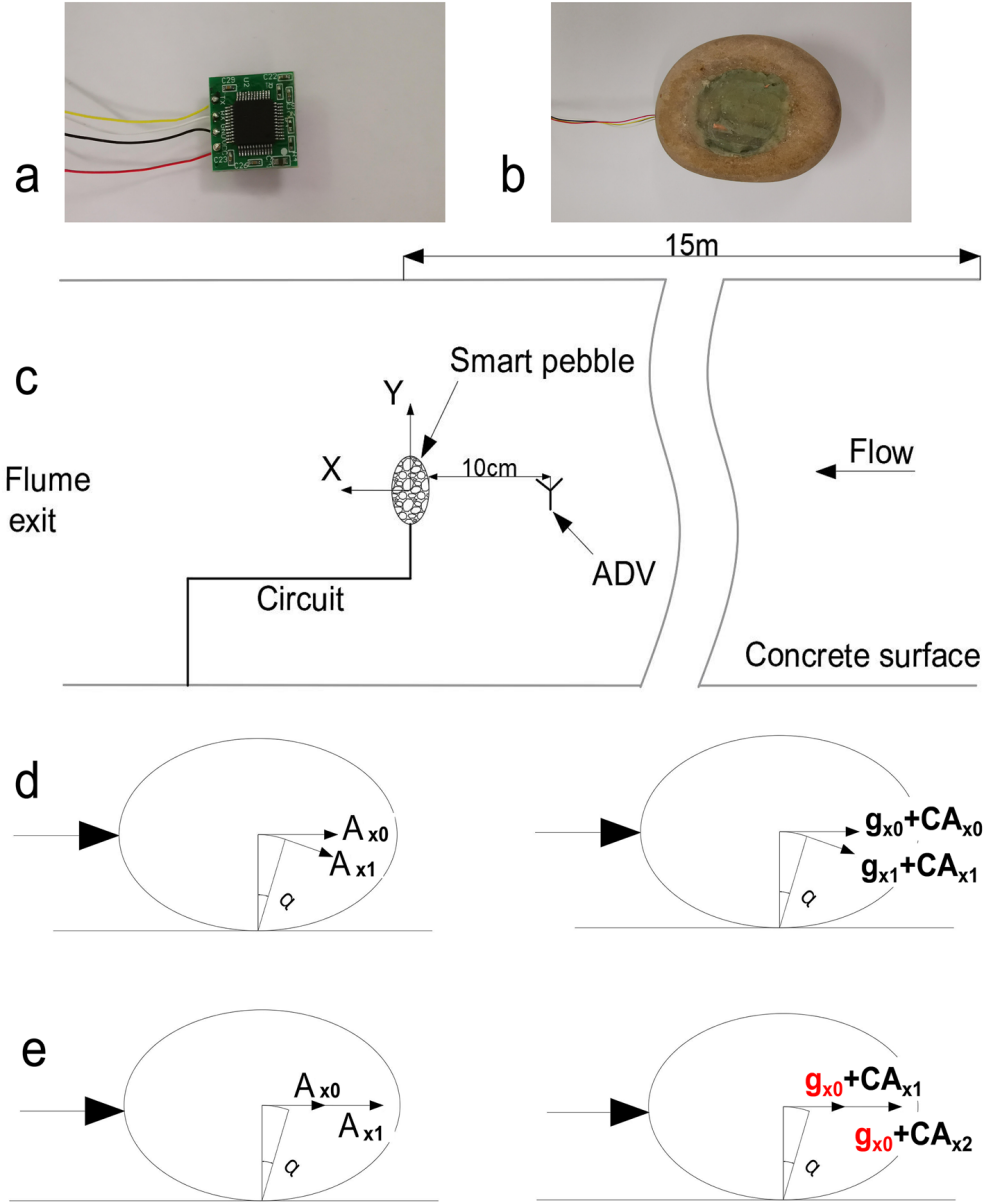
Pictures related to smart pebble test. (**a**) Miniature triaxial acceleration sensor. (**b**) Smart pebble. (**c**) A sketch of the different flume sections (top view). (**d**,**e**) Vibration process of smart pebble.

A natural pebble (length * width * height = 6.762 cm * 5.274 cm * 3.113 cm, density 2558 kg/m^3^) was selected for the test, and a 2.5 cm diameter hole was made in the middle. The sensor (Fig. 1a) is waterproofed and installed inside the pebble (0.98 cm from the bottom). In order to restore the pebble to its original capacity, the pebble was filled with fine steel wire and copper powder, and finally sealed with waterproof glue (Fig. 1b). The sensor is a customized digital output small size, industrial grade MEMS acceleration sensor with a 3.6 g measurement range, 0.1 mg resolution, 200 Hz sampling frequency, and 15 mm * 15 mm * 2.3 mm size. The coordinate arrangement of the smart pebble is shown in Fig. 1c. Considering the problem of data loss from underwater wireless transmission and the impact of sensor size, the sensor discards the hardware of battery and memory card. Smart Pebble uses a wired transmission method (Fig. 1b). To avoid wire interference with pebble movement, the sensor uses four ultra-fine Teflon high-temperature silver-plated wires of 0.35 mm diameter to complete the power supply and data transmission functions. During the test preparation period, the wires were tested for water resistance and the results were good. To prevent the wires from interfering with the pebble movement, the wires were fixed next to and downstream of the smart pebble, as shown in Fig. 1c.

### Flume experiments

Experiments were conducted in the Hydraulics Laboratory at Chongqing Jiaotong University. A glass-walled, tilting rectangular flume which was 0.55-m wide, 0.65-m deep, and 25-m long, was used. The bed slope was kept constant for all experiments, at 0.3%. Water was supplied to the flume using pumps that draw from a constant head reservoir. The flow rate is controlled by a variable speed pump. The flume was laid with a concrete bottom of the same roughness. To ensure fully developed turbulent conditions, the test section was located 15 m downstream of the flume inlet. Fully exposed, isolated smart pebble was placed in the test section, and their vibrational processes, as well as entrainment events and local flow velocities, were monitored. The local flow velocity was measured with an acoustic Doppler velocimeter (ADV) with a sampling frequency of 100 Hz, which was set 10 cm upstream along the centerline of the test particles, an arrangement illustrated in Fig. 1c.

We conducted flume tests under five uniform flow conditions to observe the movement of pebble under different flow conditions. The sediment vibration process becomes random due to the near-bed turbulence effect and the randomness of the geometric conditions of the sediment location on the bed^26,36^. Therefore, pebbles at the same flow rate were placed at five different locations for measurement, and five sets of data were collected with a collection time of 30 s. The following steps were repeated for each flow condition. First, the test flow was released for a sufficient time to measure the flow velocity in the lateral profile 10 cm upstream of the smart pebble with ADV to determine the flow as a fully developed uniform flow. Subsequently, after the accelerometer and ADV system had reached stable operation, the near-bed flow velocity and acceleration were collected simultaneously. Twenty-five sets of data were measured in sequence.

## Results

### Data pre-processing

The pebble vibration direction is mainly the water flow direction, so only the X-axis direction acceleration is analyzed in this paper. The acceleration sensor adopts advanced digital filtering technology, which can effectively reduce the measurement noise and improve the measurement accuracy, and the output data is still accurate even under the harsh environment. However, observing the original vibration signal, it is found that there are burrs on the vibration signal data curve, so the data is smoothed to reduce the impact of interference signals on the real data.

The acceleration (A_x_) output from the sensor contains both gravitational acceleration (g_x_) and vibration acceleration (CA_x_). The purpose of this study is to investigate the vibration characteristics of pebble, so the effect of gravitational acceleration data (g_x_) needs to be removed. As shown in Fig. 1d, the sensor output acceleration is $A_{{x_{0} }} \left( {A_{{x_{0} }} = g_{{x_{0} }} + CA_{{{\text{x}}_{0} }} } \right)$ at the moment of $t_{0}$,when the pebble is stationary at moment $t_{0}$, the pebble vibration acceleration $CA_{{x_{0} }} = 0$, $A_{{x_{0} }} = g_{{x_{0} }}$. The sensor output acceleration data is $A_{{x_{1} }} \left( {A_{{x_{1} }} = g_{{x_{1} }} + CA_{{{\text{x}}_{1} }} } \right)$ at the moment of $t_{0} + \Delta t$. Because the pebble vibration path (before entrainment) is circular, the gravitational acceleration of the pebble changes in both magnitude and direction during the vibration process, $g_{{x_{0} }} \ne g_{{x_{1} }}$ ($g_{{x_{0} }}$ is known, $g_{{x_{1} }}$ is unknown), so the sensor output acceleration data $A_{{x_{1} }}$ cannot be derived from $CA_{{x_{1} }}$ at $t_{0} + \Delta t$. The experiment observed that the pebble vibration angle is very small, assuming that the pebble vibrates in a straight line, as shown in Fig. 1e. The acceleration of gravity is constant at moments $t_{0}$ and $t_{0} + \Delta t$ ($g_{{x_{0} }} = g_{{x_{1} }}$), at this time $CA_{{x_{0} }} = A_{{x_{0} }} - g_{{x_{0} }}$ and $CA_{{x_{1} }} = A_{{x_{1} }} - g_{{x_{0} }}$. When the amplitude of pebble vibration increases, the $CA_{{x_{1} }}$ error increases and cannot represent the most realistic vibration data of the pebble, but still can express the vibration characteristics of the pebble. Therefore, in this paper, when analyzing the vibration characteristics, the vibration acceleration (CA_x_) is obtained by subtracting the gravitational acceleration (g_x_) from the filtered raw data (A_x_).

### Motion state identification

**Figure2:**
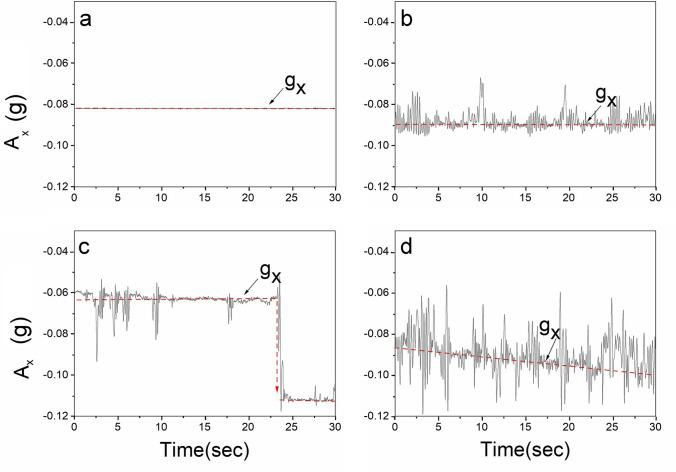
Representative time series of A_x_ (The vibration signal output by the sensor), The dotted line shows the acceleration due to gravity (g_x_), The four pictures represent different working conditions: (**a**) stationary state (v = 31 cm/s), (**b**) grain vibration (v = 34 cm/s), (**c**) sediment rolling (v = 44 cm/s), (**d**) sediment pushing (v = 44 cm/s).

Figure 2 allows to determine the state of motion of the pebble. Analyzing 25 sets of data, the test results showed that the pebble had four states of motion. As in Fig. 2a, the acceleration data is a straight line, indicating that the pebble is stationary (near-bed velocity v = 31 cm/s), and the peak local instantaneous turbulence force has not overcome the pebble resistance (mainly frictional resistance). As the flow rate increases, the vibration data are observed to fluctuate up and down around the baseline (gravitational acceleration g_x_) at the near-bottom flow rate v = 34 cm/s (Fig. 2b), which is consistent with the experimental observation of particle vibration. The pebbles vibrated because the local transient turbulence force overcame the pebble resistance, prompting the pebble to tilt in the direction of water flow, and then the transient turbulence force decreased, while the gravity of the particles prompted the pebble to return to their original position, and the inertia force caused the particles to tilt in the opposite direction, which repeatedly produced the vibration phenomenon. The essence of grain vibration lies in the fluctuation of the near-bed velocity. When the near-bed velocity was 43 cm/s (Fig. 2c), the baseline in the graph changed abruptly, indicating that the gravitational acceleration of the grain changed and it rolled. The data were consistent with the experimental observation of pebble entrainment. The rolling event is due to the average turbulent force overcoming the pebble resistance (friction and gravity), pushing the pebble to tilt substantially. When a low-frequency peak turbulent force event is encountered, the pebble destabilizes. The baseline in Fig. 2d gradually decreases, indicating a change in gravitational acceleration, consistent with the experimentally observed phenomenon of pebble slowly pushing along the downstream direction. The pushing event occurs because when the flow velocity increases, the turbulence is enhanced, and the peak turbulence force makes the pebble tilt forward, and when the pebble is tilted backward by gravity to recover its original position, it has not yet reached the original tilt limit point, and the turbulence force continues to push the pebble forward. Therefore when the particle is shaken, the vibration origin gradually changes.

The randomness of particle motion is related to the bed position where the sediment particle is located, in addition to the turbulence effect. Table 1 shows the results of the tests under five water flow conditions. The test observed that when v = 31 cm/s, the five measurement points A_x_ = 0 cm/s^2^, the pebble was stationary. When v = 34 cm/s, the pebble vibrates four times and one time is stationary, and it is determined that the pebble starts to vibrate, at which time the flow rate is the vibration threshold of the pebble. When v = 37 cm/s, 41 cm/s, five sets of data A_x_ ≠ 0 cm/s^2^, the acceleration of gravity is constant, the pebble vibration. At v = 44 cm/s, two of the five measurement points rolled and three slowly pushed, identifying particle entrainment, when the flow rate was the entrainment threshold for pebble. Tests have proven that the measurement system can accurately capture particle vibration and entrainment threshold values.

**Table 1:** Test groups and test results.

Condition number	Flow rate (L/H)	Velocity (cm/s)	Motion state
1-1,1-2,1-3,1-4,1-5	34	31	Stationary (five groups)
2-1,2-2,2-3,2-4,2-5	39	34	Vibration (four groups), stationary (one groups)
3-1,3-2,3-3,3-4,3-5	53	37	Vibration (five groups)
4-1,4-2,4-3,4-4,4-5	69	41	Vibration (five groups)
5-1,5-2,5-3,5-4,5-5	86	44	Rolling (two groups), pushing (there groups)

## Discussion

### Particle vibration categories

**Figure 3:**
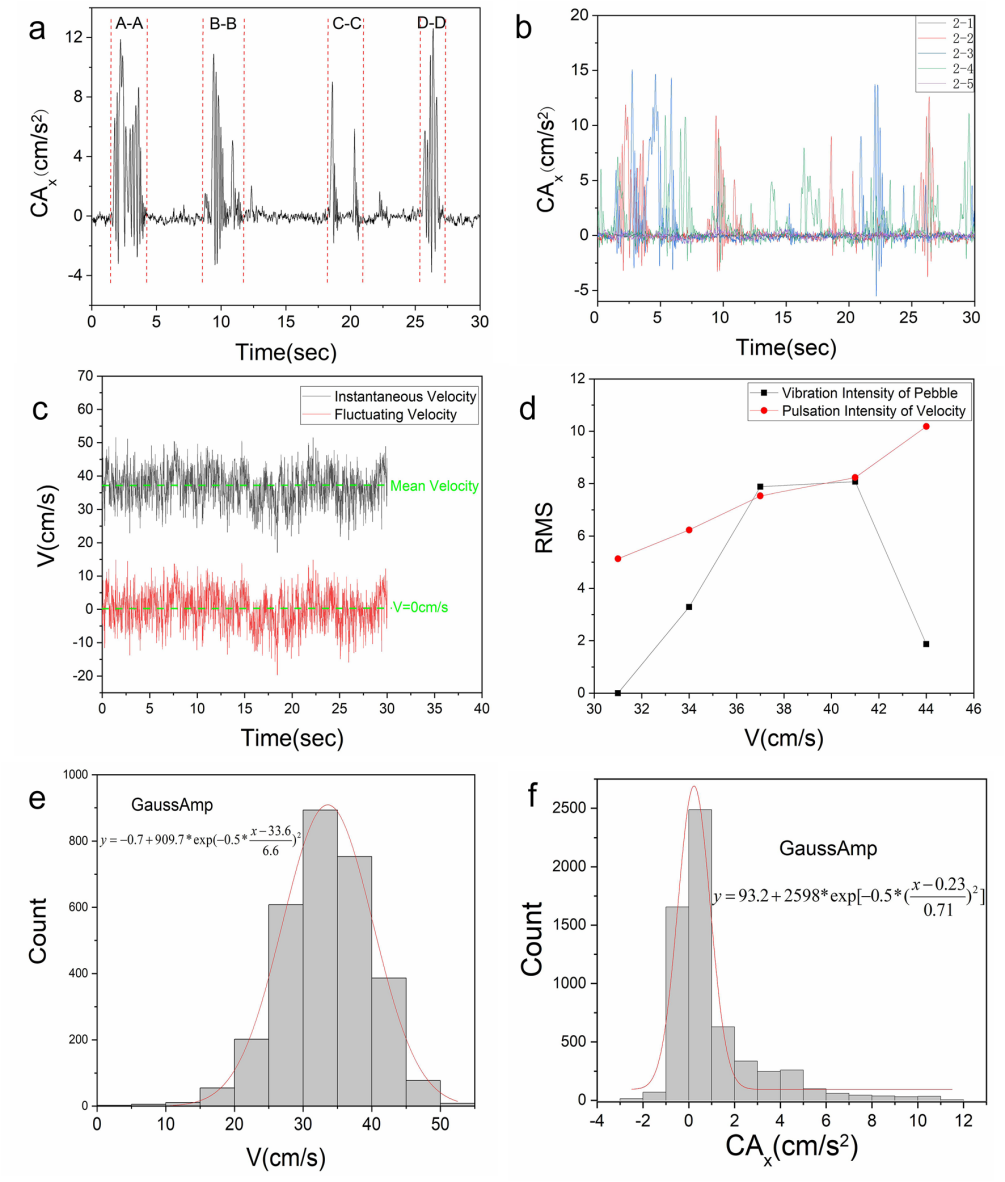
Time domain characteristic curve of pebble vibration and near-bed velocity. (**a**) Classification of pebble vibration types (Representative time series of CA_x_,The dashed range indicates the type of ectopic strong vibration). (**b**) Time course of vibration acceleration for the same flow rate and different position conditions. (**c**) The signal of fluctuating velocity and instantaneous velocity. (**d**) Vibration intensity and flow pulsation intensity versus near-bottom flow velocity. (**e**) Probability distributions of near-bed velocity. (**f**) Probability distributions of vibration acceleration (CA_x_).

Pebble vibration is excited by the water flow and produces a vibration response. According to the acceleration magnitude, the vibration type before pebble entrainment is divided into two forms: (1) in-situ vibration and (2) ectopic strong vibration (Fig. 3a). In-situ vibration is caused by turbulence that causes the pebble to sway back and forth around the origin. Ectopic strong vibration, is due to the grain meeting the high-energy turbulence event^18,37^, the vibration acceleration increases, the tilt angle becomes larger, and a strong vibration phenomenon out of the original position is produced. However, after the high-energy turbulence event is over, the pebble is subjected to gravity and returns to its original position of vibration. Due to the low frequency of high-energy turbulence events, only a few ectopic strong vibration events occur during the vibration (t = 30 s). It is observed experimentally that the high-energy turbulence events are mainly caused by the congestion effect of the flume, the influence of surface waves, and the superposition of vortices. The vibration acceleration measured in this paper reflects the impact of rapidly fluctuating hydrodynamic forces on grain vibration. It demonstrates that not all local flow velocity fluctuations above the mean value can lead to particle entrainment^15^.

### Time domain characteristics of particle vibration

Figure 3b shows that the pebble vibration varies at different locations for the same flow rate. This phenomenon proves that the pebble vibration characteristics are influenced by the bed geometry. Since random vibration is characterized by irregularity of vibration, any physical quantity of vibration cannot be expressed by a definite time function, so to further analyze the pebble vibration characteristics, this paper uses statistical indicators to analyze the time-domain characteristics of the vibration signal. The article mainly analyzes the pre-entrainment data. In the cases of Q = 39 L/h, 53 L/h, and 69 L/h, the number of samples per group N = 6000, and the acquisition time t = 30 s. In the case of Q = 39 L/h, there are only four groups of vibration conditions. In the case of Q = 86 L/h, due to the pebble entrapment event, only pre-entrapment data can be collected, the number of samples collected N = 460–2300, and the effective acquisition time t = 2.3–11.5 s.

Table 2 shows the statistical parameters of 25 sets of acceleration data, and it can be found that the statistical parameters vary under the same water flow conditions without any regularity, which again proves that the pebble vibration is a random phenomenon influenced by the bed position. Table 2 shows that of the 25 sets of mean acceleration data, 24 groups are greater than zero, and 1 set is less than zero, indicating that the pebble vibrate mainly in the direction of the water flow under the impact of the current, and a few cases in the opposite direction of the water flow. The latter event was due to the raised contact surface between the pebble and the riverbed, which prevented the pebble from vibrating forward. To overcome the randomness caused by the bed shape and to analyze the pattern of statistical parameters with flow rate, the parameters under the same flow conditions are averaged in this paper. From the mean data, it can be seen that before approaching the threshold value, the mean value of mean squared difference and maximum value tends to increase, and the mean value of minimum value decreases as the flow increases, indicating that the discrete degree of pebble vibration acceleration is enhanced. However, near the threshold value, the mean values of mean squared difference and maximum value decrease, and the mean value of minimum value increases. The mean values of skewness coefficients were more significant than 1 for different water flow conditions, indicating that the probability distribution graph of vibration acceleration was shifted to the left.

**Table 2:** Time domain statistical parameters of pebble vibration acceleration.

	Average value	Mean square error	Skewness coefficient	Maximum value	Minimum value
Condition number (Q = 39 L/h)					
2-1	0.58	2.14	3.09	12.61	− 3.78
2-2	0.69	2.55	3.32	15.09	− 5.52
2-3	− 3.27	8.64	− 0.55	17.80	− 26.52
2-4	1.04	1.99	2.26	11.12	− 2.45
Average value	− 0.19	3.08	1.62	11.40	− 7.73
Condition number (Q = 53 L/h)					
3-1	1.32	5.70	3.61	40.39	− 19.56
3-2	8.38	12.45	0.68	45.53	− 23.11
3-3	1.67	3.98	1.79	22.56	− 5.54
3-4	4.93	9.20	1.59	50.05	− 23.69
3-5	1.36	3.52	2.53	20.22	− 5.07
Average value	3.53	6.97	2.04	35.75	− 15.39
Condition number (Q = 69 L/h)					
4-1	0.35	4.53	− 0.40	26.36	− 26.11
4-2	7.68	17.36	0.79	91.94	− 83.73
4-3	0.24	3.52	2.84	22.89	− 15.90
4-4	2.37	7.26	1.33	33.83	− 27.32
4-5	2.30	5.16	2.14	25.95	− 7.70
Average value	2.59	7.57	1.34	40.20	− 32.15
Condition number (Q = 83 L/h)					
5-1	2.41	3.56	− 0.06	13.29	− 7.58
5-2	0.60	7.59	0.45	33.18	− 23.64
5-3	0.99	0.88	0.06	4.45	− 1.48
5-4	0.15	1.10	1.16	5.00	− 3.05
5-5	0.13	1.80	3.67	16.32	− 6.45
Average value	0.86	2.99	1.05	14.45	− 8.44

As the pebble vibration is a random phenomenon, the vibration intensity of the same water flow condition was averaged to analyze the variation of pebble vibration intensity at different flow rates. Root mean square value analysis is a common data analysis method for signal processing, which mainly analyzes the average effective energy of the signal, and its expression is

1 $$ A_{rms} = \sqrt {\frac{1}{N}\sum\limits_{i = 1}^{N} {X_{i}^{2} } } $$

where ${\mathrm{X}}_{\mathrm{i}}$ is the vibration acceleration value, and N is the number of vibration acceleration samples.

In order to analyze the pulsation intensity variation law of the flow velocity, the near-bed flow velocity signal is considered as the sum of the average flow velocity and the pulsation flow velocity, as shown in Fig. 3c. The root-mean-square value of the pulsating flow velocity is taken as the pulsation intensity of the flow velocity. As shown in Fig. 3d, before approaching the threshold value (v = 44 cm/s), the pulsation intensity of the flow velocity increased with the increase of the flow velocity, and the pebble vibration intensity also increased, however, the vibration intensity weakened when approaching the threshold value. Because when approaching the entrainment threshold, the turbulent force generated by the mean flow velocity is greater than the particle resistance. It pushes the particles to tilt, at which time the pebble lack the inertial force to restore their original position and can only vibrate slightly under the action of the current pulsation force. When the high-energy turbulence event occurs, the pebble is entrained. This result is in agreement with Williams' observation of solid particles^38^.

The near-bed velocity is the main water flow parameter that determines whether the sediment vibrates and the intensity of the vibration. Observing the PDF plot of the instantaneous flow velocity near the bottom (Fig. 3e), it was found that it approximately obeyed a normal distribution (consistent with the findings of related studies). The pebble vibration event inherits the randomness of turbulent fluctuations, and the vibration acceleration PDF plot (Fig. 3f) before pebble entrainment is observed to conform to a normal distribution function. It belongs to the normal distribution with large kurtosis and sloping to the left. The results indicate that the pebble vibration is strongly correlated with the current action. The probabilistic model is characterized by a left near-Gaussian function and a long right tail. The acceleration in the near Gaussian part is relatively small and is mainly caused by in-situ vibrational events. The long right tail describes the ectopic strong vibrational events, so it is most relevant to high-energy turbulence events.

### Spectral characteristics of particle vibration

The frequency domain analysis reflects the distribution of vibration energy and the components of vibration frequency. For this purpose, the acceleration data (X(t)) need to be converted to a complex function (Z(w)) in the frequency domain through the Fourier transform, the mathematical formula is

2 $$ Z\left( \omega \right) = \frac{1}{2\pi }\int_{ - \infty }^{\infty } {X(t)e^{ - j\omega t} } dt $$

**Figure 4:**
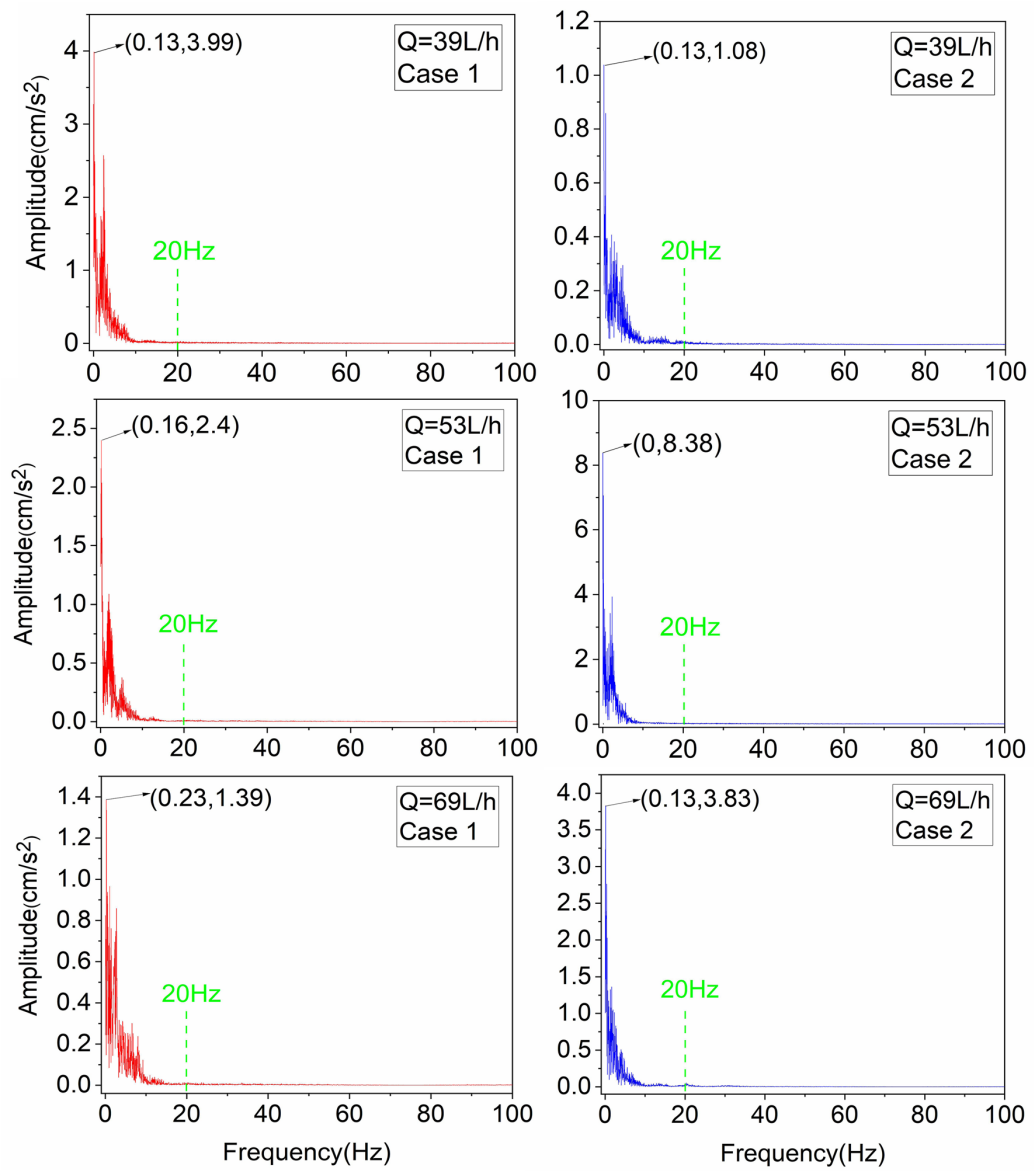
Frequency spectrum of pebble vibration acceleration at different flow rate. The pictures represent different working conditions: Q = 39 L/h, Q = 53 L/h, Q = 69 L/h.

The working conditions of the spectrum analysis are consistent with those explored in the time domain, all of which are the vibration data before entrainment. Among them, the effective data of the five working conditions at Q = 86 L/h are less and different, so the accuracy of the analysis is weak compared with other working conditions. However, to analyze the vibration characteristics of the pebble near the entrainment threshold, this paper makes an approximation to analyze the frequency distribution under this water flow condition, and the results show that the vibration frequency is concentrated in the range of 25 Hz. Figure 4 shows the pebble vibration spectrum curves. It can be found that 98% of the energy of the pebble vibration signal is concentrated in the range of 20 Hz, indicating that the pebble vibration signal is a low-frequency signal. The data show that the pebble vibration intensity decreases but the vibration frequency increases when approaching the entrainment threshold. Observing Fig. 4, it was found that the amplitude maximum was concentrated within 0.5 Hz, which was influenced by the high-energy turbulence event.

**Figure 5:**
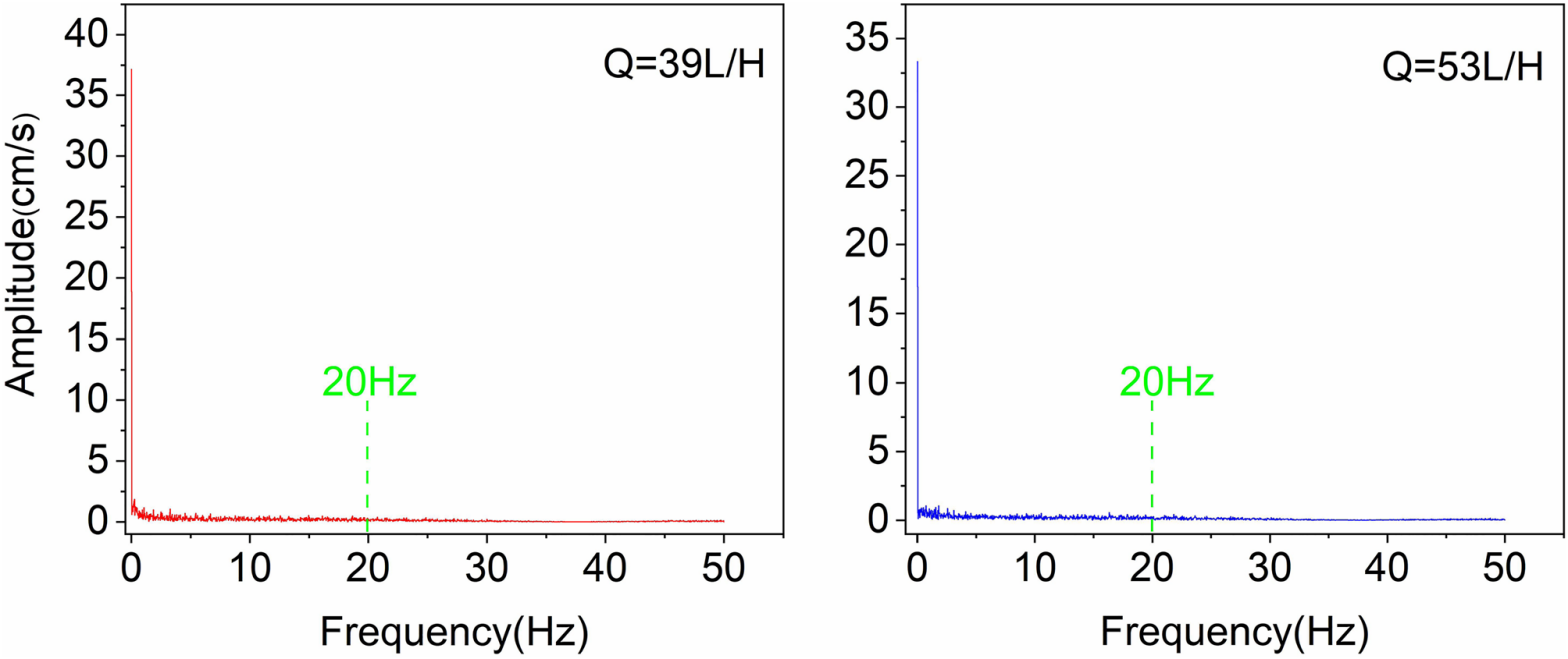
Frequency spectrum of near-bed velocity at different flow rate.

The near-bed flow velocity was converted from the time domain to the frequency domain (Fig. 5). It is found that 97% of the energy of the flow velocity signal is concentrated within 20 Hz. The results indicate that the near-bed flow velocity signal is a low frequency signal^39^. Therefore, both the pebble vibration acceleration and the flow velocity are low-frequency signals with similar frequencies. The pebble is excited by the water flow to produce a vibration response, and vibrate according to the frequency of the excitation signal, in line with the pebble vibration mechanism, proving that the data measured by the measurement system are reliable.

## Conclusions

Based on the phenomenon of vibration or swaying before sediment particle entrainment, a micro inertial accelerometer was used for the first time to measure the vibration process of fully exposed, isolated natural pebble on a rough bed surface and to collect the near-bed velocity simultaneously. In this paper, a series of experimental studies with different water flow conditions were conducted. This study is the first attempt to collect vibrational acceleration data of sediment particles and analyze them in conjunction with near-bed turbulence data, and the main findings are as follows,

1. The smart pebble designed in this paper is capable of collecting the vibration acceleration before entrainment, and measuring the event process of particle entrainment close to the initial motion flow conditions. The data show results consistent with the experimentally observed motion, proving that the system is effective in measuring pebble nonlinear vibrations.
2. Grain vibration (before entrainment) is divided into two types: (a) in-situ vibration; (b) ectopic strong vibration, the former because the pebble is excited by turbulent forces and generates a vibration response, and the latter because a high-energy turbulent event in the turbulent flow generates strong vibration. The data demonstrate that high-energy turbulence events have a more significant effect on sediment vibration and are the dominant factor in pebble entrainment. The conclusions follow a similar theoretical framework as done by Valyrakis et al.^18^.
3. The probability distribution (PDF) of the pebble vibration acceleration (before entrainment) conforms to the normal distribution, inheriting the randomness of turbulent fluctuations, indicating the significant kinetic significance of local turbulence in pebble vibration.
4. The pebble vibration intensity, before approaching the threshold value, tends to increase, and the intensity weakens when approaching the threshold value.
5. The pebble vibration frequency is within 20–25 Hz, similar to the flow pulsation frequency. It is subject to high-energy turbulent events with amplitude maxima concentrated within 0.5 Hz, indicating that the near-bed velocity is most directly related to particle vibration events.

## Data Availability

The datasets generated and/or analysed during the current study are not publicly available due to ongoing and confidential nature but are available from the corresponding author on reasonable request.
